# The association of maternal nutrition and children’s pre-primary experience with over-age attendance in secondary school: evidence from lowland Nepal

**DOI:** 10.1016/j.ijer.2019.10.001

**Published:** 2020

**Authors:** Akanksha A. Marphatia, Jonathan C.K. Wells, Alice M. Reid, Mario Cortina Borja, Dharma S. Manandhar, Aman Sen, Naomi Saville, Delan Devakumar, David Osrin, Audrey Prost, Sophiya Dulal

**Affiliations:** a Department of Geography, University of Cambridge, Cambridge, UK; b Institute of Child Health, University College London, UK; c Mother and Infant Research Activities (MIRA), Kathmandu, Nepal; d Suaahara II, Helen Keller International, Chakupat, Lalitpur, Nepal; e Institute for Global Health, University College London, UK

## Abstract

• Over-age attendance is increasing but remains under-studied in South Asia.
• Children fall behind by entering pre-primary or primary late, and by repeating a grade during/after primary school.
• Rural location, thin and uneducated mothers predicted late pre-primary entry.
• Educational research and interventions need to focus on the earlier time-point of pre-primary.
• Improving maternal nutrition and education may ensure timely progression of children in school.

## 1 Introduction

Lower educational attainment contributes to cycles of disadvantage, which propagate poverty, poor nutritional status and lack of opportunity across generations (UNESCO, 2017). Although this linkage between education and broader human capital and health outcomes is widely recognised (UNDP, 2018), efforts have primarily focused on increasing the number of children in school. However, many who attend school may be ‘silently excluded’ from achieving their full learning potential because of poor attendance and low achievement (Lewin, 2007). This is a crucial issue to address because it ultimately increases the risk of early drop-out from school (Nonoyama-Tarumi, Loaiza, & Engle, 2010; Sunny et al., 2017; UIS & UNICEF, 2015; Wils, 2004).

Another key underlying reason for this silent exclusion is slow progression in school, or faltering in grade level, referred to in this article as ‘grade slippage.’ This may occur due to entering primary school at an older than the official recommended age and/or due to grade repetition (Lewin, 2007). These factors collectively manifest most clearly as over-age attendance, defined as being ≥2 years older than the expected age for a given grade (UNESCO, 2017). The magnitude of over-age attendance is substantial across low-income countries (LICs) where, in 2015, 25% were over-age in primary and 43% in secondary school (UNESCO, 2017). However, whilst awareness of this common problem is increasing, as shown by its inclusion as a thematic indicator in the fourth Sustainable Development Goal (SDG) (UNESCO UIS, 2017), there are several gaps in research.

First, most research on over-age attendance has addressed primary school (Hungi, Ngware, & Abuya, 2014; Jha & Lewin, 2014; Nonoyama-Tarumi et al., 2010; Sunny et al., 2017; Wils, 2004). Starting primary school late usually means being over-age throughout one’s educational career, and often further slippage. In China, for example, a one-year delay in primary school enrolment increased the risk of first-grade repetition by 10% and reduced the odds of secondary school enrolment by 6% (Chen, 2015). Whilst this research has emphasized the development of over-age as a process, it may be ignoring the most important stages in the life-course, the years *prior* to primary school. Pre-primary education is a relatively recent development in LICs, but its availability is rapidly increasing (UNESCO, 2017), and it needs to be better incorporated in educational and broader human capital research.

Second, while the overall value of attending pre-primary school is accepted, little attention has been directed to its association with subsequent over-age attendance. Children failing to gain critical developmental and social skills early in life have less opportunity to acquire them at later ages (Black et al., 2016; Engle et al., 2011; Jaganath, Khatry, Murray-Kolb, LeClerq, & Christian, 2015). However, starting late and spending more years in pre-primary results in entering primary school late and staying over-age/grade with little potential benefit (Black et al., 2016). For example, a study from Uruguay found only small additive benefits in the educational attainment of children at age 12 years after attending two years of pre-primary, and no gains for spending three years (Berlinski, Galiani, & Gertler, 2006). We therefore need to investigate how variability in pre-primary experience may impact subsequent educational trajectories. To our knowledge, few studies have collected data on both the age of entry and duration of pre-primary.

Third, since educational disparities begin early and intensify over time (Rose & Alcott, 2015), we need to investigate the factors shaping pre-primary entry-age. For example, for both late primary entry and lower educational attainment, a range of risk factors have been identified. These include physical barriers to school access (e.g. poor transport or adverse weather) (Ayral, 2014), low household wealth (Neupane, 2017; Nonoyama-Tarumi et al., 2010), poor parental education (Jha & Lewin, 2014; Nonoyama-Tarumi et al., 2010; Seshie-Nasser & Oduro, 2016; Shrestha & Shrestha, 2017), poor infant growth (Adair et al., 2013; Brown & Pollitt, 1996; Fentiman, Hall, & Bundy, 2001; Glewwe & Jacoby, 1993; Martorell et al., 2010) and maternal undernutrition (Marphatia, Devakumar et al., 2016). However, the impact of these factors may vary by children’s level of schooling (Glewwe & Jacoby, 1995; Halpern & Myers, 1986), and we lack understanding of how they may shape pre-school experience.

Fourth, existing research on over-age attendance is geographically uneven. Most evidence on the penalties of over-age attendance comes from studies in Africa (Hungi et al., 2014; Seshie-Nasser & Oduro, 2016; Sunny et al., 2017; Wils, 2004). Paradoxically, very few studies directly focus on this issue in South Asia, a region where these problems are most acute and endemic (Alcott & Rose, 2017; Hossain, 2010; Moock & Leslie, 1986; Sabates, Hossain, & Lewin, 2013). For example, in Nepal, where our study is based, 36% of primary and 43% of lower secondary school students were over-age for their grade in 2015 (UNESCO, 2017). These data suggest that grade slippage may begin early on, in the pre-primary years, and may relate to broader developmental factors.

To address these research gaps, this paper seeks to contribute new understanding on the independent association of pre-primary experience and grade repetition with over-age attendance in secondary school. Since starting school late invariably means being behind for the rest of one’s schooling, an investigation of the factors shaping late pre-primary entry is crucial. Given the young age at which children start pre-primary schooling, factors acting in the period prior to school entry are important to consider. Using a socio-ecological conceptual model, we investigate the underlying maternal biological and household socio-economic factors associated with late pre-primary entry.

Empirically, we use longitudinal data from a double-blind Randomised Controlled Trial (RCT) of antenatal micronutrient supplementation of 797 mother-child dyads from lowland Nepal to test the following hypotheses:

1. Among children who are over-age for their grade in secondary school, this grade slippage begins early, by starting pre-primary school late
2. Maternal undernutrition and lack of education, independent of low socio-economic status, are associated with late pre-primary entry
3. Poor infant growth trajectories mediate the association of maternal undernutrition and pre-primary entry-age

### 1.1 Research context

Our study was conducted in Dhanusha district, in the lowland Terai region of Nepal, which has a population of 768,000, 50% of whom are women (Ministry of Health & Population Nepal, New ERA, ICF International, 2012). In 2016, 17% of women aged 15-49 years were thin (Body Mass Index, BMI of below 18.5 kg/m^2^) and 41% anaemic; 36% of children under five were stunted (low-height-for-age) and 27% underweight (low-weight-for-age) (Ministry of Health & Population, Nepal, New ERA, ICF International, 2017). Approximately 33% of women aged 15-49 years were uneducated, 6% had completed primary grade five and 10% had completed secondary grade 10 (Ministry of Health & Population et al., 2017).

The basic education system in Nepal provides one year of early childhood education and development (ECED) or pre-primary, eight years of primary, and three years of secondary school (Ministry of Education Nepal, 2016). In parallel, community-based ECED centres (managed mostly by non-governmental or private organisations) also offer three years of early learning (nursery, lower and upper kindergarten) (Jaganath et al., 2015; Ministry of Education Nepal, 2009). Children are expected to enter pre-primary at the age of three years and primary grade one at the age of five years (UNESCO, 2017).

In the Terai, from 2007-2009, when our cohort was in pre-primary school, the overall Gross Enrolment Ratio (GER, total number of students enrolled in pre-primary, irrespective of age, expressed as a percentage of the official school-age population corresponding to the same level of education) was 64%, with 55% of children entering primary grade 1 having ECED experience in 2007 (Government of Nepal, 2009). In 2009, 87% of pre-primary schools were public (fee-free) and 13% were private (fee-paying) (Government of Nepal, 2014a). Many early learning centres do not achieve minimum quality standards because of inadequate physical infrastructure, learning materials and age- and language-appropriate curricula (Cumming, Acharya, Moriani, Shakya, & Levine, 2012; Khanal, Paudyal, & Dangal, 2017). Across Nepal in 2007, 73% of ECED teachers were trained, but training is often of poor quality and teacher turnover is high (Khanal et al., 2017; UNESCO, 2010). Public institutions generally have one facilitator per class compared to two in private schools, suggesting that children may not receive relevant and individual attention (Government of Nepal, 2014a; Khanal et al., 2017), especially the 1.1% with learning disabilities in primary school (Government of Nepal, 2009).

In primary grade one, a significant proportion of children were already older than the expected entry-age of five years, suggesting they either started this level or pre-primary late, or spent more time in pre-primary. This early faltering affects subsequent over-age enrolment, as shown by a GER exceeding 100%. In 2009-2010, when our cohort would have been attending primary grade one, the GER was 142% (UIS, 2018). Fewer girls than boys were enrolled in pre-primary (GER 64% vs 67% in 2009) but more girls than boys were enrolled in primary (GER 141% vs. 138% in 2009-2010) and secondary (GER 72% vs 67% in 2015-2016) (UIS, 2018).

## 2 Conceptual model

**Fig. 1:**
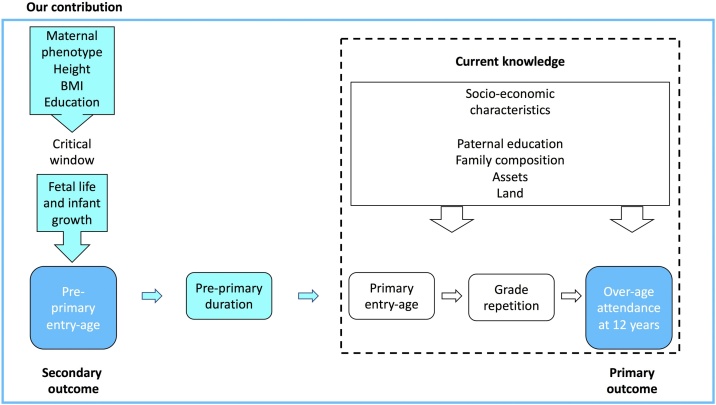
Socio-ecological conceptual model: variables used in analyses.

Fig. 1 illustrates the socio-ecological conceptual model we used to investigate the association of multiple factors with two outcomes (shown in darker shaded boxes): over-age attendance (primary outcome) and late pre-primary entry (secondary outcome).

Current research (shown in boxes within dashed line)focuses on how socio-economic factors, primary entry-age and subsequent grade repetition shape over-age attendance. Our contribution is shown in the shaded boxes to the left of the dashed line. We extend the current framework by including pre-primary school, both in terms of entry-age and duration. To investigate the pathways shaping the timing of entry into this initial learning stage, we include not only child growth trajectories, which are used by other studies to examine lower educational attainment in general (Adair et al., 2013; Maluccio et al., 2009; Martorell et al., 2010), but also the maternal factors which shape them in the first place (Wells, 2010).

The focus on maternal ‘phenotype,’ a biological term referring to the sum total of observable traits that characterise any individual, is important because early child growth occurs within the context of the unique physiological niche provided by mothers during an early ‘critical window’ of development: pregnancy and early infancy. Previous studies have found that maternal phenotype exerts greater influence on early child growth than external environmental factors acting in later periods (Lucas, 1991; Wells, 2010). Proxies for maternal somatic traits include height, a marker of health status accumulated through genetic influences and environmental exposures in early childhood, and Body Mass Index (BMI or weight/height^2^), which reflects current nutritional status (Özaltin, Hill, & Subramanian, 2010). These traits are associated with children’s cognitive and educational outcomes through, for example, maternal nutritional transfer during fetal and brain development in the last weeks of pregnancy (Marphatia, Reid, & Yajnik, 2019; MacKay, Smith, Dobbie, & Pell, 2010; Marphatia, Devakumar et al., 2016; Stein et al., 2013). The collective magnitude of these maternal traits represents what is ultimately passed onto offspring (Schell, 1997; Smith, Ramakrishnan, Ndiaye, Haddad, & Martorell, 2003).

Household factors provide the broader social context within which maternal factors shape child outcomes (Reed, Habicht, & Niameogo, 1996). Both material assets and land ownership (especially for those living in rural areas) are proxies for broader income, labour and food security (Kafle, Jolliffe, & Winter-Nelson, 2018; Moock & Leslie, 1986). Parental education represents both the value and investment attributed to children’s education and development, with mothers and fathers potentially providing differential support (Alderman & Headey, 2017; Lloyd & Blanc, 1996; Sabates et al., 2013).

The ways in which these pathways both shape children’s educational trajectories and are also shaped by education illustrates the integrated nature of the SDGs. We hypothesise that factors acting outside of school, and potentially in the years prior to school entry or before children are even born, may be the most significant predictors of early grade faltering.

## 3 Materials and method

### 3.1 Study profile

We used data from a double-blind Randomised Controlled Trial (RCT) of antenatal micronutrient supplementation (Devakumar et al., 2014; Dulal et al., 2018; Osrin et al., 2005; Vaidya et al., 2008). Research ethics approvals for the RCT and subsequent follow-ups were granted by the Nepal Health Research Council (reference 16/2015) and the ethics committee of the University College London Institute of Child Health and Great Ormond Street Hospital for Children (reference 6513/001), London, UK. This study is registered as an International Standard Randomised Controlled Trial, number ISRCTN88625934. We obtained consent (signed, or witnessed thumbprints from those who were illiterate) from pregnant women for their participation in the RCT at recruitment (Osrin et al., 2005) and at follow-ups: verbal consent at 2.5 years and signed consent at 8.5 and 12 years from parents or guardians of children (Devakumar et al., 2014; Dulal et al., 2018; Osrin et al., 2005; Vaidya et al., 2008). At the 12-year follow-up, we also obtained verbal assent from children for their participation in the study (Dulal et al., 2018).

**Fig. A1:**
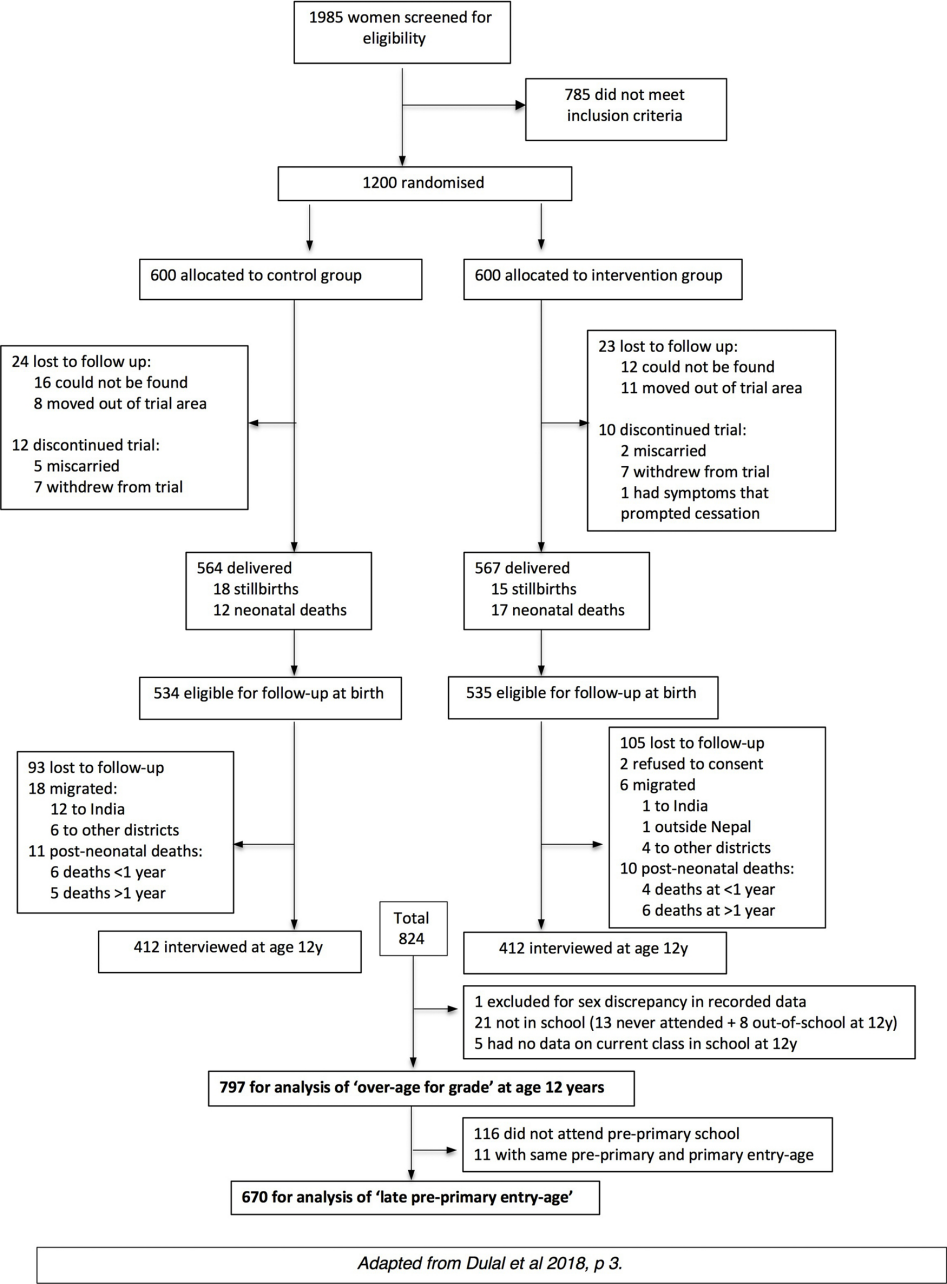
Study profile. This flowchart describes follow-up rates and exclusions at recruitment, birth, and ages 2.5, 8.5 and 12 years. Although we separate by trial intervention and control groups, our study analyses all children, and controls for trial allocation groups in logistic regression models.

Between 2002-2004, 1200 mothers were recruited through the antenatal care clinic at the Janakpur Zonal Hospital and follow-ups were conducted by home visits by trained fieldworkers. Women were excluded from the trial if gestation was beyond 20 weeks, they had multiple pregnancies, fetal abnormalities, existing illness that could compromise the pregnancy’s outcome, or if their residence was potentially inaccessible by fieldworkers (Osrin et al., 2005). Appendix Fig. A1 lists exclusion criteria for follow-ups.

Women were randomly allocated to receive either a daily multivitamin and mineral supplement, or a control supplement of iron and folic acid (Osrin et al., 2005). In total, 1069 mothers completed the trial. The main trial outcomes were duration of gestation; child weight and size at birth and at ages 2.5, 8.5 and 12 years (Devakumar et al., 2014; Dulal et al., 2018; Vaidya et al., 2008); and overall intelligence and executive function at 12 years (Dulal et al., 2018).

Data on maternal traits and household characteristics (including size of land owned) were collected prospectively by questionnaire at enrolment into the study (mean gestation of 15 weeks of pregnancy), and are described elsewhere (Osrin et al., 2005). Data on children’s schooling years completed were collected at age 8.5 years and 12 years (Dulal et al., 2018; Marphatia, Devakumar et al., 2016). At the 12-year follow-up in 2015-2016, further data on children’s entry-age and age-related participation and progression in education from pre-primary onwards were collected retrospectively by oral questionnaire administered to mothers (Dulal et al., 2018). Fathers were also asked how many years of schooling they had completed.

To ensure the meaning of pre-primary school was understood by respondents, during the piloting of questionnaires, we specifically asked mothers about their child’s pre-primary attendance, including whether s/he had attended a nursery, lower and/or upper kindergarten, in case these terms were more familiar. We also discussed the age at which their child started the different levels of schooling, and how many years they spent at each level (including repetition). The reliability of these data was checked against the child’s exact date of birth (collected in the RCT at birth), and by charting their progression in school and failure by grade level and by age. In some cases when mothers were unsure about their children’s schooling, we also asked the child’s father.

### 3.2 Variables

#### 3.2.1 Outcome variables

Our primary outcome variable, ‘over-age attendance’, was dichotomised as ‘expected/higher vs. lower’ grade at the mean age of 12 years. We defined this variable based on the grade children were attending and their age. The exact age of children was recorded by the trial and at subsequent follow-ups. At the recent follow-up, children’s age ranged from 10-13 years, with a mean of 12 years.

**Fig. 2:**
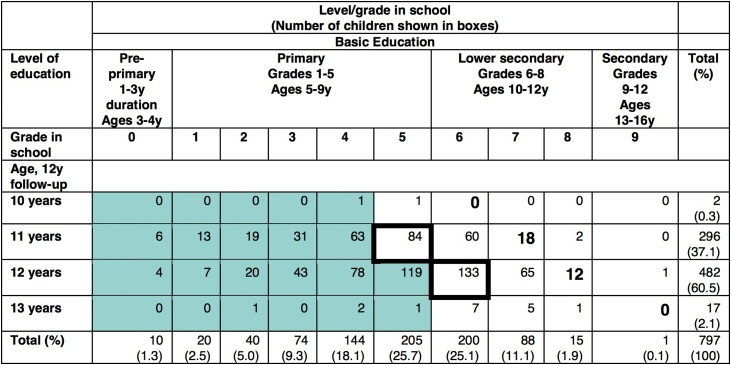
Distribution of children by age and grade, early adolescence.

To calculate ‘over-age attendance,’ we undertook several steps. According to the structure of the Nepalese education system, at ages 10-13 years, children should be attending grades six to nine (Ministry of Education Nepal, 2016; UNESCO, 2017). However, this ‘official age/grade categorisation’ did not reflect the empirical reality of our cohort, or previous research (Ministry of Education Nepal, UNICEF, & UNESCO, 2016; Moock & Leslie, 1986). Fig. 2 shows the internal distribution of our cohort by age and grade according to the structure of the education system in Nepal. It was most common for an 11-year-old child in our cohort to be in grade level five (*n=*84), and a 12-year-old child to be in grade six (*n=*133) (boxes outlined in black). Children were generally attending a grade below the most common grades (shaded, to the left of boxes outlined in black) than at or above it (unshaded, to the right of boxes outlined in black). On average, children in our cohort increased educational attainment by less than a complete grade with each successive year of age. Overall, we see that our cohort was attending a lower grade for their age in comparison to the ‘official age/grade’ classification (noted in larger bold font).

Using this internal distribution of our cohort’s grade by age, we coded children in ≥5 grade as being in the expected/higher grade for age 11 years and similarly, those in ≥6 grade for age 12 years. Children in a lower grade than expected for age were coded as follows: ≤4 grade for age 11 years and ≤5 grade for age 12 years. The outer two years of age, 10 and 13, contained very few children. We therefore grouped together the threshold for expected/higher vs. lower grade for age as the same for 10 and 11 years, and for 12 and 13 years.

Overall, there is a general lack of precision, or difficulty in measuring over-age attendance, be it in our survey or government cut-offs. Unless a child’s birthday is on the date of the exact start of the school year, children will most likely move through two ages during the school year. Furthermore, if the survey on schooling status is conducted at the beginning of the school year, more children will be younger, and if the survey is towards the end of the school year, then more children will be older. These factors may partly explain that whilst for 11-year olds in our cohort the most common grade level was five, there were still 60 children attending grade six. By creating our cut-off at grade five for 11-year olds, we have partly addressed this issue. The underestimation of over-age children in national data (Ministry of Education Nepal et al., 2016; Moock & Leslie, 1986) may also explain the discrepancy with our results. A greater proportion of over-age children live in rural areas, in the far western development region and poorest households (Ministry of Education Nepal et al., 2016).

Our second outcome variable, ‘late pre-primary entry’, was dichotomised as ‘expected/younger vs. older’ age. The entry-age of our cohort into pre-primary did not match the official national entry-age of three years (UNESCO, 2017). We therefore coded this variable according to the distribution in our cohort, with expected/young entry-age of ≤4 years and older entry-age of ≥5 years.

#### 3.2.2 Potential predictors of over-age attendance at age 12 years

Potential predictors of over-age attendance included pre-primary entry-age (y) and duration (y), primary entry-age (y) and grade repetition (yes or no).

To include children who had not attended pre-primary school and also to account for the difference in duration of different types of pre-primary school, we coded the variable years of pre-primary school completed as: not attended (0-1y), attended a 1-year government school, attended 1-3 years private pre-primary, or repeated pre-primary (government or private).

Primary school entry-age was coded in relation to the distribution of our cohort rather than the official national entry-age of five years (UNESCO, 2017). This approach resulted in an expected/younger primary entry-age of ≤7 years vs. older entry-age of ≥8 years. We limited the variable of grade repetition to primary school onwards because repetition at the earlier time-point was already reflected in years of pre-primary completed.

#### 3.2.3 Potential predictors of pre-primary entry-age

Potential predictors of pre-primary entry-age included maternal traits and household socio-economic characteristics collected at recruitment into the RCT. Data on paternal educational attainment were collected at the 12-year follow-up. We also included children’s early growth trajectories, from birth to age 2.5 years.

Maternal somatic traits, measured during pregnancy, at recruitment into the trial, included age (y), parity (number of births prior to the index child), blood haemoglobin level (g/dl), stature (cm), BMI (kg/m^2^), and duration of pregnancy (weeks). Maternal height was categorised in relation to the distribution of our cohort, as short (≤146.9 cm), average (147 to 153 cm) or tall (≥153.1 cm). Maternal BMI was categorised as low (≤18.50 kg/m^2^), average (18.51 to 20.49 kg/m^2^), or high (≥20.50 kg/m^2^) based on cut-offs used for South Asian populations (WHO Expert Consultation, 2004). Maternal haemoglobin levels were coded as anaemic (≤11.00 g/dl) or non-anaemic (≥11.10 g/dl) according to World Health Organization (WHO) classification (WHO, 2011). Parity was used as a categorical variable (no births previous to the index child, 1-2 or ≥3 children). Duration of pregnancy was coded according to the clinical definition of pre-term (≤37 weeks) vs. term (>37 weeks) (March of Dimes, PMNCH, Save the Children, & WHO, 2012).

Education of both parents was coded into three levels as per the classification used by Nepal’s education system: none; primary/lower secondary (basic education) (1-8y); or secondary and higher (≥9y) (Ministry of Education Nepal, 2016; UNESCO, 2017). Due to small numbers, we did not further disaggregate by year of schooling. We also included household location (urban or rural), material assets (‘high, mid or low’ based on predefined criteria from WHO (Devakumar et al., 2014; WHO SEARO, 1994) and size of agricultural landholding (continuous value, in area using local units, 1 dhur = 0.004 acres or 3.6 square feet) owned by families.

We used measures for children’s weight (kg) and height (cm) at birth and at the age of 2.5 years. We also included early growth, which by convention is assessed relative to a reference population, for age and sex. We first converted children’s weight (kg) and height (cm) at birth and at age 2.5 years into age-and sex-specific *z*-scores (SDS) using WHO reference data (which do not adjust for gestational age) (Cole and Green (1992). Then we computed conditional *z*-scores for these variables at age 2.5 years, adjusting for size at birth, to give indices of early postnatal growth (Adair et al., 2013). These were calculated as the standardised residuals of the regression of height at 2 years on height at birth, to assess the extent to which the child followed the population growth trajectory or whether they exceeded or fell below it at 2 .5 years. By taking into account both the relationship of current size with size at birth, and regression to the mean between birth and 2.5 years, these scores provide a numerical value for growth trajectory in early life (Adair et al., 2013).

### 3.3 Statistical analysis

Maternal BMI and agricultural land were right skewed and therefore natural log-transformed for statistical tests, but reported in the original scales in Tables. We used the continuous value of conditional growth in height and weight between birth and the age of 2.5 years in our statistical tests. We also reported the proportion stunted (<-2 SD), underweight (<-2 SD) and wasted at age 2.5 years (<-2 SD) because they are easier to interpret (WHO and UNICEF, 2009). A scatterplot of continuous values of children’s primary against pre-primary entry-ages in months allowed assessing the functional relationship between both entry ages.

Categorical variables were compared using chi-squared tests and continuous variables using independent samples *t*-tests. We also tested for correlations (Pearson’s coefficient) between maternal and family components using continuous values for these variables. There were small numbers of missing values with primary entry-age having the largest relative frequency of missing values (*n* = 52, or 6.5%) for individual variables. We used the complete datasets for every analysis.

Our logistic regression models quantified the relative risk, expressed in Odds Ratios (OR) with 95% Confidence Interval (CI), of (a) over-age attendance at the age of 12 years and (b) late pre-primary entry-age. Variables that were not associated with the outcome in univariable (having only one predictor) models were not retained for multivariable (having several predictors) analyses. Nagelkerke’s statistic (NK), a pseudo *R*^2^ measure, was multiplied by 100 to show the proportion of variance explained in over-age attendance and pre-primary entry-age. We retained in models variables with association *p* < 0.1.

We tested all continuous variables for linear association with the outcomes. The reference group was set as the highest level of the predictor (e.g. maternal secondary educational attainment). For children's sex, we set girls as the reference group because analysis by chi-squared test showed a greater proportion of boys were over-age/grade. Although there were no significant gender differences in pre-primary entry-age, we maintained girls as the reference group to maintain consistency with the over-age attendance model. We controlled for children’s age and sex, and for trial allocation group. We introduced sex interaction terms to explore additional risk of predictor variables for boys' over-age attendance.

All analyses were conducted in SPSS 24 (IBM Corp.).

## 4 Results

### 4.1 Summary statistics

#### 4.1.1 Lost to follow-up vs. retained at age 12 years

Appendix Fig. A1 shows that, at the 12-year follow-up, 824 children were interviewed. For this analysis, 27 additional children were excluded: one child had a discrepancy in sex in the recorded data, 21 were out-of-school (described below), and five had no data on the current class they were attending in school.

The total sample for our primary outcome - ‘over-age attendance at age 12 years’ - was 797 children (indicated in bold text in Appendix Fig. A1). These 797 children represented a retention rate of 74.5% of the 1069 children recruited at birth.

Our secondary outcome, ‘late pre-primary entry-age,’ comprised 670 children (indicated in bold text in Appendix Fig. A1). For this analysis, 127 additional children were excluded: 116 had not attended pre-primary school (described below) and 11 had the same entry-age recorded for both pre-primary and primary school. The 670 children included in our secondary outcome represented a retention rate of 62.7% of the 1069 children recruited at birth.

In comparison to the children who had attended pre-primary school, a greater proportion of the 116 who had not attended pre-primary were from larger households with lower socio-economic capital: 1-2 children (45% vs. 51%, *p* ≤ 0.001), uneducated parents (43% vs. 78% of mothers and 13% vs. 46% of fathers had no schooling, *p* ≤ 0.001), fewer assets (12% vs. 28%, *p* ≤ 0.001) and less land (31% vs 40%, *p* ≤ 0.05). In comparison to those who attended pre-primary, those who had not attended were also more likely to reside in rural areas (56% vs. 73%, *p* ≤ 0.001), come from the Dalit plains (2% vs 4%, *p* ≤ 0.001) or other Terai (68% vs. 74%, *p* ≤ 0.001) ethnic groups and follow the Muslim (4% vs. 12%, *p* ≤ 0.01) religion.

Additional analyses showed that of the 21 children out-of-school, 13 had never been to school and eight had dropped out by the age of 12 years. These 21 children tended to come from disadvantaged households: 95% had uneducated mothers, 62% had uneducated fathers, 33% had thin and 38% short mothers, 43% were from low asset households, and 52% of children were underweight, 5% wasted and 24% stunted at 2 years. About 71% were from rural areas. Grade faltering began early for the eight children who had dropped out: 75% had not attended pre-primary, and those who had, started late; all had repeated a standard and dropped out by grade three. Four girls left school because families could not afford it, or to help at home, due to distance, or because they were not interacting with others. Four boys left school due to unaffordability, illness, because education was not considered important, or due to poor performance.

Summary statistics in Table 1 show small differences between the 403 children lost to follow-up and 797 included in our study.

**Table 1:** Summary statistics, maternal phenotype and socio-economic characteristics, lost to follow-up vs retained at 12-years.

	**1**: Lost to follow-up (*n=*403)		**2**: Followed-up at 12y (*n=*797)		**3**: Difference^1^		
	**Mean**	**SD**	**Mean**	**SD**	**Δ**	**s.e.**	***p-*value**
Maternal age (y)	21.5	3.7	21.5	3.5	0.03	0.2	0.903
Land owned (dhur) (*n=*1132)^3^	4.8	9.0	4.7	8.7	1.0	1.1	0.886
Birthweight of child (kg) (*n=*1052)	2.7	0.5	2.8	0.4	−0.04	0.03	0.117
	***n***	**%**	***n***	**%**		***p*-value**^2^	
**Maternal phenotype**							
Maternal height (*n = *1197)						0.716	
Short (≤146.9 cm)	99	24.6	179	22.5			
Average (147.0 to 153.0 cm)	170	42.3	346	43.5			
Tall (≥153.1 cm)	133	33.1	270	34.0			
Maternal education						0.054	
None	163	40.4	381	47.8			
Primary/lower secondary (1-8y)	97	24.1	168	21.1			
Secondary+ (≥9y)	143	35.5	248	31.1			
Maternal parity						0.664	
0	187	46.4	353	44.3			
1-2	173	42.9	364	45.7			
≥3	43	10.7	80	10.0			
Maternal BMI (*n=*1197)							
Low (≤18.50 kg/m^2^)	103	25.6	240	30.2		0.211	
Average (18.51-20.49 kg/m^2^)	155	38.6	300	37.7			
High (≥20.50 kg/m^2^)	144	35.8	255	32.1			
Maternal anaemia (*n=*1172)						0.578	
Anaemic (≤11.00 g/dl)	152	39.8	301	38.1			
Not anaemic (≥11.10 g/dl)	230	60.2	489	61.9			
Birth gestation (*n=*1139)						**0.001**	
Pre-term (≤37 weeks)	51	14.9	50	6.3			
Term (>37 weeks)	291	85.1	747	93.7			
**Socio-economic characteristics**							
Residence (*n=*851)						0.414	
Rural	50	63.3	452	58.5			
Urban	29	36.7	320	41.5			
District						0.663	
Dhanusha	333	82.6	655	82.2			
Mahottari	67	16.6	139	17.4			
Sarlahi	1	0.2	2	0.3			
Siraha	2	0.5	1	0.1			
Ethnic origin						**0.016**	
Dalit plains	11	2.7	20	2.5			
Muslim	34	8.4	43	5.4			
Janjati hills	16	4.0	16	2.0			
Other Terai (plains) groups	238	59.1	548	68.8			
Brahmin Chhetri hills	28	6.9	44	5.5			
Brahmin Chhetri plains	76	18.9	126	15.8			
Religion						**0.003**	
Hindu	364	90.3	754	94.6			
Muslim	34	8.4	42	5.3			
Other	5	1.2	1	0.1			
Material assets (score) (*n=*1199)						0.120	
Low	74	18.4	111	13.9			
Medium	127	31.6	274	34.4			
High	201	50.0	412	51.7			
Paternal education (*n=*803)						na	
None	na	na	137	17.5			
Primary/lower secondary (1-8y)			174	22.2			
Secondary+ (≥9y)			472	60.3			

Abbreviations: SD, standard deviation. Difference lost to follow-up relative to followed-up. s.e., standard error. *n*, number.

1 Independent Samples *t-*test.

2 Chi-squared test.

3 Natural log-transformed; untransformed value reported.

#### 4.1.2 Maternal and socio-economic characteristics

Table 1, Column 2 shows maternal and family traits of our sample. Around a third of mothers were of poor nutritional status, proxied by short stature, low BMI and anaemia. Nearly half of mothers were uneducated compared to 17% of fathers. Most households were Hindu and of the Terai plains or Brahmin Chhetri plains ethnic origin. About 14% the families had low assets, and 58% resided in rural areas, mostly in Dhanusha district.

#### 4.1.3 Child characteristics

Table 2 presents summary statistics of children’s growth trajectories. As expected, given that they are standardised residuals, the average conditional growth values were zero, and the standard deviations were ∼1. At the age of 2.5 years, 41% of our cohort were stunted, 62% were underweight and 8% were wasted.

**Table 2:** Summary statistics, child growth and educational trajectories.

	Mean	SD
**Children’s growth trajectories**		
Conditional linear growth, birth to 2.5 years (*n =* 762)	0.02	0.99
Conditional weight-gain, birth to 2.5 years (*n = *766)	0.00	1.00

	***n***	**%**
Stunted at age 2.5 years (<-2 SD) (*n = *764)		
Yes	316	41.4
No	448	58.6
Underweight at age 2.5 years (<-2 SD) (*n = *764)		
Yes	474	62.0
No	290	38.0
Wasting at age 2.5 years (<-2SD) (*n = *764)		
Yes	61	8.0
No	703	92.0
**Children’s educational trajectories**		
Pre-primary attended (*n = *681)		
Yes	681	85.4
No	116	14.6
Pre-primary years completed		
Not attended (0-1 year)	123	15.4
Attended 1-year government school	47	5.9
Attended 1-3 years private school	502	63.0
Repeated either government or private school	125	15.7
Primary entry-age (*n = *745)		
Expected/younger, ≤7 years	482	64.7
Older, ≥8 years	263	35.3
Type of primary school attended (*n = *787)		
Private	511	64.9
Government	190	24.1
Both private and government	86	10.9
Type of lower secondary school attended (*n = *303)		
Private	154	50.8
Government	147	48.5
Both private and government	2	0.7
Grade repetition, primary onwards		
Yes	89	11.2
No	708	88.8

Abbreviations: SD., standard deviation. *n*, number. Number of cases for variables are noted only if <full sample size of 797.

Table 2 also shows children’s educational trajectories. About 85% of our cohort had attended pre-primary school, which is substantially higher than the regional Terai estimate (64%) (Government of Nepal, 2009). Over half (63%) of our cohort attended a private pre-primary school. Attendance in private primary was 65% and lower secondary school 51%.

About 16% had repeated pre-primary school, and 35% were already over-age upon entering primary school. Other studies, and our own qualitative data, indicate that multiple years spent at pre-primary may be explained by parents preferring children to ‘repeat’ in three-year private institutions, even after having completed a one-year government nursery because private schools are perceived to provide better education than public ones (Jaganath et al., 2015). About 11% had repeated a grade in or after primary.

### 4.2 Outcome variables

We report outcome variables in the text below rather than in a Table.

#### 4.2.1 Primary outcome: over-age attendance at age 12 years

At a mean age of 12 years, 408 (51.2%) children were over-age for their grade vs. 389 (48.8%) at the expected/younger grade/age. A greater proportion of boys (57.7%) than girls (44.4%) were over-age for their grade (*p* <  0.001).

#### 4.2.2 Secondary outcome: late pre-primary entry-age

Just under a third of children (198: 29.6%) had entered pre-primary school at an older age (≥5 years) vs. 472 (70.4%) at the expected or younger age of 4 years. There were no differences by child sex in pre-primary entry-age (p 0.451), with 28.1% of girls and 30.8% of boys starting pre-primary late.

### 4.3 Predictors of over-age attendance at age 12 years

#### 4.3.1 Univariable analyses

Univariable models showed pre-primary duration (years completed) was associated with over-age attendance. Relative to not attending pre-primary school, attending a 1-year government pre-primary was not associated with over-age attendance (Odds Ratio, OR 1.24, 95% CI 0.62, 2.48), but attending 1-3 years in a private pre-primary (OR 1.57, 95% CI 1.04, 2.37) and repeating this initial level of education (OR 4.19, 95% CI 2.43, 7.23) increased the risk of being over-age for grade at age 12 years. However, subsequent analysis demonstrated the number of years spent at pre-primary was confounded by pre-primary entry-age. This could be because a greater proportion of the children who spent more time in pre-primary (>3 years) started pre-primary at a younger age of ≤4 years.

Primary entry-age, grade repetition from primary onwards and pre-primary entry-age were associated with over-age attendance and retained in subsequent multivariable analyses. We still included pre-primary duration in models to show that it was not a significant predictor of over-age attendance at 12 years once other factors were included.

#### 4.3.2 Multivariable analyses

In Table 3, we first tested whether primary entry-age (Model 1) and grade repetition from primary onwards (Model 2) were independently associated with over-age attendance. These models reflected current knowledge on the factors associated with over-age attendance (Brunette et al., 2017; Glewwe & Jacoby, 1993; Moock & Leslie, 1986; Nonoyama-Tarumi et al., 2010; Seshie-Nasser & Oduro, 2016; Wils, 2004). Our contribution comes from additionally including pre-primary experience, both in terms of entry-age and duration (Model 3), and also testing the relative importance of each predictor for over-age attendance at the age of 12 years (Model 4).

**Table 3:** Multivariable logistic regression models of school entry-age and progression with over-age attendance at mean age 12 years.

	**Current knowledge of predictors**				**New contribution of pre-primary experience**			
	1: Primary entry-age		2: Grade repetition		3: Pre-primary experience		4: Pre-primary, primary, repetition	
	(*n=*745)^1^		(*n=*786)^2^		(*n=*670)^3^		(*n=*635)^4^	
	NK=0.272		NK=0.062		NK=0.235		NK=0.356	
	**OR**	**95% CI**	**OR**	**95% CI**	**OR**	**95% CI**	**OR**	**95% CI**
Children’s age (at 12-year follow-up)	**1.43**	1.05, 1.93*	**1.34**	1.02, 1.77*	1.31	0.95, 1.80	1.29	0.91, 1.84
Trial allocation arm (Control = Ref)	1.00		1.00		1.00		1.00	
Micronutrient Supplementation	1.10	0.79, 1.53	0.88	0.66, 1.17	1.19	0.85, 1.68	1.33	0.91, 1.94
Child’s sex (Girl = Ref)	1.00		1.00		1.00		1.00	
Boy	**1.55**	1.12, 2.14**	**1.74**	1.30, 2.32***	**1.69**	1.20, 2.37**	**1.61**	1.11, 2.33*
Primary entry-age (≤ 7y = Ref)	1.00						1.00	
Older, ≥ 8 years	**8.24**	5.69, 11.94***					**5.14**	3.29, 8.01***
Grade repetition (No = Ref)			1.00				1.00	
Yes, ≥ primary school			**2.89**	1.74, 4.79***			**4.19**	2.04, 8.62***
Pre-primary entry-age (≤4y = Ref)					1.00		1.00	
Older, ≥5 years					**6.43**	4.24, 9.75***	**2.99**	1.79, 5.00***
Pre-primary duration (none = Ref)					1.00		1.00	
1y government school					0.40	0.60, 2.58	0.58	0.07, 4.96
1-3y private school					0.75	0.13, 4.34	0.90	0.12, 6.98
Repeated (gvt or private)					2.75	0.45, 16.68	2.26	0.28, 18.46
Constant	0.01*		0.02*		**0.02**		**0.01**	

Abbreviations: SD., standard deviation. n, number. Number of cases for variables are noted only if < full sample size of 797.

1 Sample size (*n=*381 below expected grade for age, *n=*364 expected/higher grade for age).

2 Sample size (n=401 below expected grade for age, *n=*385 expected/higher grade for age).

3 Sample size (*n=*309 below expected grade for age, *n=*361 expected/higher grade for age).

4 Sample size (*n=*344 below expected grade for age, *n=*291 expected/higher grade for age).

* *p*≤0.05.

** *p*≤0.01.

*** *p*≤0.001.

Table 3 shows that primary entry-age (Model 1) and grade repetition (Model 2) were both independently associated with over-age attendance at age 12 years. Both models show that boys were more at risk of being over-age/grade than girls.

The small proportion of children (11%) who repeated a primary or lower secondary grade may also partly explain the lower effect size of this variable on over-age attendance at the age of 12 years. The proportion of the variance explained by late primary entry-age (27.2%) was much greater than that explained by grade repetition (6.2%), suggesting that starting school late had a greater effect than subsequent grade slippage on over-age attendance at 12 years.

Table 3, Model 3 included all children who had attended pre-primary school and those with information on pre-primary experience and primary entry-age, so it would be comparable with Models 1, 2 and 4. Results show that boys were more at risk of being over-age for their grade than girls. Late pre-primary entry-age predicted over-age attendance. Spending more time in pre-primary was not associated with over-age attendance once entry-age was controlled for. As previously mentioned, this may be because a greater proportion of the children who spent more time in pre-primary (>3 years) started pre-primary at a younger age of ≤4 years. This model explained 23.5% of the variance in over-age attendance.

The slightly lower proportion of the variance explained by pre-primary Model 3 in comparison with the primary entry-age Model 1 (27.2%) may be explained by the fact that not all children attended pre-primary, and some experienced further grade slippage after primary school. However, preliminary analyses found that not attending/spending less than one year in pre-primary was not associated with over-age attendance. Although these children missed out on early learning, the majority (95.5%) started primary school before or around the expected age of seven years.

Table 3, Model 4 included all previous predictors: primary entry-age, repetition, and pre-primary entry-age and duration, to show the independent contribution of these factors. Boys were more likely to be over-age for their grade. Late primary entry-age, grade repetition from primary onwards and late pre-primary entry-age were independently associated with over-age attendance.

In comparison to Models 1, 2 and 3, the magnitude of the association with child sex did not change in Model 4. The coefficients for late primary and pre-primary entry-age decreased, suggesting that these variables may be correlated, although they each still contributed independently to being over-age for grade. The coefficient for repetition from primary school onwards increased. Late entry and repetition are likely to be alternative routes to being over-age. For example, we did not find that the children who started pre-primary or primary late were more likely to repeat a grade level after primary school (which applied to only 11% in our cohort). In comparison to Models 1, 2 and 3, final adjusted Model 4 explained a greater proportion of the variance, 35.6%, in over-age attendance. We found no effect of antenatal micronutrient supplementation on over-age attendance.

**Fig. 3:**
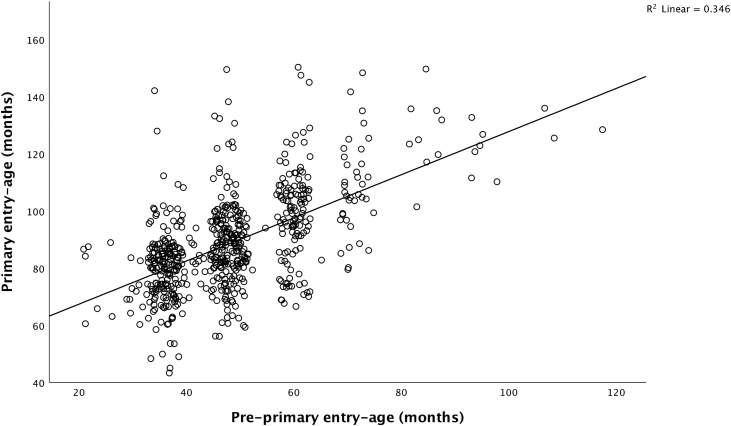
Scatterplot, pre-primary entry-age and primary entry-age.

In Model 4, primary entry-age had a greater magnitude of the effect than pre-primary entry-age and when both were included in the Model, the coefficients for both decreased, suggesting they may be correlated. To further explore this result, we produced a scatterplot of these two variables in Fig. 3. Whilst entering pre-primary late will invariably delay primary entry-age, the scatterplot shows a moderately weak correlation (R^2^ =0.346) between these two variables, which is partly explained by the larger variance in the primary age-entry values for pre-primary entry values above 65 months. Factors other than the age at entry at pre-primary may also account for later primary entry-age. These may include spending more years in pre-primary school, or reflect factors acting outside of school such as maternal undernutrition, low household SES, lower parental engagement or access to psychosocial stimulation.

Overall, these results support our first hypothesis, showing that over-age attendance in lower secondary school reflects starting pre-primary school at a later age. Hence, starting late is enough on its own to make people over-age for their grade throughout their schooling career. Repeating a grade after primary school was also associated with being over-age for grade. We found that boys were more likely than girls to be over-age for their grade at age 12 years. Given the importance of the timing of entry into this initial stage of schooling for subsequent and sustained grade slippage, we investigate the predictors of entering pre-primary school at a later age.

### 4.4 Predictors of late pre-primary entry-age

#### 4.4.1 Univariable analyses

Maternal age, anaemia status and pre-term birth were not predictors of late pre-primary entry in univariable logistic regression models and were not carried forward to subsequent analyses.

Univariable models showed some variables were associated with pre-primary late entry, but subsequent models showed they were ultimately confounded by other factors. For example, relative to a mother who had no previous children, if the index child had older siblings [1-2 siblings (OR 1.35, 95% CI 0.95, 1.93, p 0.094) and ≥3 siblings (OR 2.02, 95% CI 1.12, 3.64)], they were likely to enter pre-primary later, but this was because both late entry and larger families were shaped by having mothers of lower educational attainment.

Similarly, households with lower land ownership (OR 1.72, 95% CI 1.06, 2.81) were likely to have children who entered pre-primary late, but this was because of lower material assets. In turn, low (OR 3.13, 95% CI 1.88, 5.21) and mid-level assets (OR 1.96, 95% CI 1.35, 2.83) were shaped both by lower maternal educational attainment and rural location. Given these results, we discarded parity and material assets and retained maternal education and rural location in further models.

Correlations between maternal and household traits may partly explain confounding of the above associations (Appendix Table A1). For example, the negative correlation between maternal education and parity (-0.30) suggests more schooling to be related to fewer children. In communities that participated in our study, where women generally marry before having children, women who have stayed in school longer would more likely marry at a later age, and thus have less time to have more children. The correlation between land ownership and material assets (0.21) suggest both may be proxies of household wealth, which may differ in rural areas. In our study, maternal education confounded the association of assets with pre-primary entry-age. The correlation between material assets and maternal educational attainment (0.46) suggests that the latter may be a proxy for SES, with better educated mothers more likely to marry into families with greater wealth.

#### 4.4.2 Multivariable analyses

Table 4, Model 1 shows that shorter, uneducated, lower educated, thinner mothers and rural location independently increased the risk of entering pre-primary at an older age. Antenatal micronutrient supplementation protected against late pre-primary entry. This model explained 18.9% of the variance in pre-primary entry-age.

**Table 4:** Multivariable logistic regression models of predictors of late pre-primary entry-age.

	**1: Early-life factors** (*n=*645)^1^ NK=0.189		**2: Early-life/child growth** (*n=*615)^2^ NK=0.228	
	**OR**	**95% CI**	**OR**	**95% CI**
Child’s age	0.83	0.58, 1.18	1.12	0.74. 1.68
Allocation (Control = Ref)	1.00		1.00	
Micronutrient supplementation	**0.66**	0.46, 0.95*	**0.66**	0.45, 0.97*
Child’s sex (Girl = Ref)	1.00		1.00	
Boy	1.01	0.70, 1.47	1.26	0.85, 1.88
Maternal height (Tall, ≥ 153.1 cm = Ref)	1.00			
Short (≤ 146.9 cm)	**1.94**	1.17, 3.19**	1.59	0.94, 2.70^3^
Average (147–153 cm)	1.13	0.74, 1.74	0.91	0.58, 1.44
Maternal education (Secondary, ≥ 9y = Ref)	1.00			
None	**3.59**	2.27, 5.69***	**2.95**	1.79, 4.85***
Primary/lower secondary (1–8 y)	**1.80**	1.05, 3.11*	1.63	0.92, 2.91^4^
Maternal BMI (High, ≥ 20.50 kg/m^2^ = Ref)	1.00			
Low (≤18.50 kg/m^2^)	**1.66**	1.03, 2.66*	1.61	0.97, 2.65^5^
Average (18.49–20.49 kg/m^2^)	1.41	0.90, 2.20	1.45	0.91, 2.32
Location (urban = Ref)	1.00			
Rural	**2.72**	1.84, 4.02***	**2.83**	1.88, 4.27***
Children’s conditional linear growth, birth to 2y			**0.62**	0.49, 0.80***
Constant	0.72		0.02	

Abbreviations, OR= Odds Ratio, 95% CI = Confidence Interval, *n=* Number included in analysis, NK= Nagelkerke, pseudo r^2^.

1 Sample size (*n=*451 young/expected entry-age, *n=*194 older entry-age).

2 Sample size (*n=*432 young/expected entry-age, *n=*183 older entry-age).

3 p=0.084.

4 p=0.096.

5 p=0.063.

* p≤0.05.

** p≤0.01.

*** p≤0.001.

These results provide support for our second hypothesis, i.e. that both biological and social pathways directly related to mothers, independent of paternal education and SES, shape the timing of children’s pre-primary entry.

Table 4, Model 2 adds children’s conditional linear growth from birth to age 2 years into Model 1, showing that greater linear growth was protective against late pre-primary entry. This variable partially mediated the effects of maternal shorter stature and low BMI, which did not reach statistical significance. Maternal lack of education and rural residence independently predicted late pre-primary entry. Antenatal micronutrient supplementation protected against late pre-primary entry. Other than the coefficient for maternal lack of education, which decreased with the inclusion of children’s conditional linear growth at 2 years, other coefficients changed only slightly between two Models. Model 2 explained 22.8% of the variance in pre-primary entry-age.

These findings provide partial support for our third hypothesis. Whilst the coefficients for maternal stature and BMI did not differ with the inclusion of child growth trajectories, their independent association with pre-primary entry-age did not reach statistical significance. This is partly expected given the later point in time of child growth than maternal stature and BMI.

#### 4.4.3 Additional analyses

We found the association of low maternal BMI with late pre-primary entry was mediated by paternal lack of education (OR 4.70, 95% CI 2.86, 7.73) and primary or lower secondary education (OR 2.54, 95% CI 1.67, 3.86). However, this does not mean maternal BMI was no longer predictive of pre-primary late entry, rather that its effect was shown through paternal education. This may be because men with lower education may marry wives with low BMI or less education, but the husband’s education produces late pre-primary entry. Albeit weak, the correlation between maternal BMI and husband’s education (0.08) may also indicate fewer resources to support adequate maternal nutritional intake. We did not include paternal education in our models since children’s pre-primary entry-age is likely to be more directly affected by maternal BMI, which is crucial in shaping the phenotype of offspring, and a deeper driver of children’s early growth.

Although we did not find a significant effect of child sex on pre-primary entry-age, since boys were more likely to be over-age/grade, we introduced sex interaction terms for each predictor. Relative to taller mothers, boys of shorter (OR 3.26, 95% CI 1.24, 8.55) and average stature (OR 2.85, 95% CI 1.15, 7.10) mothers had an additional risk of entering pre-primary late. As shown in Table 4, infant linear growth did not entirely mediate the longer-term effect of maternal undernutrition on children’s education.

## 5 Discussion

### 5.1 Summary of key findings

In this paper, we set out to describe the different ways children in lowland Nepal were ‘silently excluded’ in school and to identify predictive factors. Whereas most studies on over-age attendance have focused on primary entry-age and subsequent grade repetition, our unique contribution was to include pre-primary entry-age and duration as potential factors. Given the importance of pre-primary experience for subsequent over-age attendance in school, we also investigated the broader factors associated with later pre-primary entry. Our results support our conceptual model, showing that maternal biological and social traits shape children’s educational trajectories. The five key findings from our analysis are briefly discussed below.

First, a higher proportion of our cohort (51%) were over-age for their grade in lower secondary school in comparison to the national average rate (43%) (UNESCO, 2017). This is concerning because our internal cut-offs for over-age attendance suggest the age-for-grade level standards established by the government are not being met. Over-age attendance remains a pressing policy challenge with geographic variation in school progression across Nepal.

Second, we found that boys were more likely to have fallen behind by secondary school, but there were no gender differences in pre-primary entry-age. Whilst we did not set out to formally test for gender differences, our findings reflect current national data, suggesting that if given a chance, girls tend to outperform boys in school (UNESCO, 2017). However, it is important to note that previous studies from Nepal find a female disadvantage in educational attainment (Ashby, 1985; Moock & Leslie, 1986; Stash & Hannum, 2001).

Third, we found that the process of falling behind started *prior* to primary education, in the pre-primary years, and initiated a domino effect. As a consequence, what happens in primary and subsequent schooling is strongly imprinted by experience in the earlier stages of schooling, especially late pre-primary entry. Whilst a greater duration of pre-primary invariably delays subsequent entry into primary school, some of the children who spent more time in pre-primary entered at a younger age. As yet, the benefit of additional years at this initial stage are unclear, with few studies examining this aspect (Berlinski et al., 2006). These erratic entry and progression patterns are thus best understood together, using longitudinal data.

Fourth, grade repetition was an additional risk factor for over-age attendance. Children who started late generally find it more difficult to ‘catch up’ on learning, especially if they did not gain foundational skills in pre-primary (Berlinski et al., 2006; Jaganath et al., 2015; Research Centre for Educational Innovation and Development (CERID) (2012); Upreti, 2016; Hammond, Linton, Smink, & Drew, 2007). For example, we found the children who had dropped out before completing primary school had not attended pre-primary, or started late and subsequently repeated a grade. The high rates of grade repetition, in especially grade one (21% in Dhanusha district in 2014-2015) (Government of Nepal, 2014a) requires greater attention.

Fifth, we have shown that maternal biological and social factors matter for children’s education. It is not just that uneducated mothers may be less able to support their children’s school progression. The effect of maternal education may partly come from early marriage, with lower educated mothers tending to marry young (Raj, McDougal, Silverman, & Rusch, 2014). Although better infant growth (size) may send social signals for readiness for school (Brown & Pollitt, 1996), it did not entirely mediate the independent effects of maternal undernutrition on children’s education. However, antenatal micronutrient supplementation, which marginally increased children’s birthweight and weight at 2.5 years (Osrin et al., 2005; Vaidya et al., 2008) protected against late pre-primary entry but did not affect over-age attendance. It is possible that, by 12 years, other health, nutrition and educational influences (e.g. late entry-age and grade repetition) overwhelmed any effects of antenatal supplementation on growth and education outcomes. A systematic review of antenatal multiple micronutrient trials with follow-up periods ranging from 0 to 9 years found no evidence that supplementation improved childhood survival, growth, or cognitive outcomes (Devakumar et al., 2016).

### 5.2 Research and policy implications

Our results inform debate on how policy and research can maximise educational attainment in low- and middle-income settings, where over-age attendance remains an endemic challenge (UNESCO, 2017).

First, given the importance of pre-primary entry-age and duration for subsequent progression in school, educational research must begin at this earlier time point, and adopt a broader ecological framework to identify predictive factors acting in early-life. Pre-primary policy interventions need to expand access, fee-free provision and improve quality so children attain foundational skills that better prepare them for primary grade one and reduce their risk of subsequent grade repetition. Consistency between the varying durations and quality of pre-primary offered by different providers may ensure age-appropriate entry and progression, as would a clear policy on ‘repetition’ and changing between public and private institutions.

Second, further research and evaluation of the liberal promotion policy in Nepal, introduced in 1999 in grades one to three and since extended to grade seven, is required (World Bank, 1999); its continuation is unclear, as it is not mentioned in the current National School Sector Plan (Ministry of Education Nepal, 2016). The policy established a minimum level of learning for each grade with continuous assessment and support to move children to the next upper grade level, partly by awarding extra points in the year-end examinations, to go over the 40% pass threshold (Government of Nepal, 2007; Sharma, 2016; World Bank, 1999). Evaluations find the policy has contributed to reducing the repetition rate and improving the transition rate from primary to lower secondary (Independent Evaluation Group, 2015; Sharma, 2016). However, placing children in a higher grade than their achievement level leads to further grade slippage and eventual drop-out, only at the later time-point, as shown by the low secondary school completion rate (Ezaki, 2019; Sharma, 2016; UNESCO, 2017). Furthermore, the substantial proportion of children who are over-age for their grade across Nepal and in our cohort suggests the promotion policy has had limited success in moving children through the school system at the expected age. Our results, like other studies, suggest that age at entry (at both pre-primary and primary levels), which occurs before any promotion policy takes effect, also contributes to over-age attendance (Hungi et al., 2014; Nonoyama-Tarumi et al., 2010; UNESCO UIS, 2017; Wils, 2004). Research using longitudinal data is needed to better understand these complex progression patterns.

Third, concerted efforts are needed to address the broad range of factors associated with high repetition rates. These include learning disabilities and undernutrition, both of which are associated with poor cognitive and learning outcomes (Fink & Rockers, 2014; Government of Nepal, 2014a; Sandjaja, Rojroonwasinkul, Nyugen, Budiman, & Ng, 2013). Household chores, employment (Neupane, 2017), erratic attendance, including temporary drop-out/re-enrolment and grade level shifts due to transfers between public to/from private schools (owing to household perception of private institutions offering better quality education) also contribute to over-age attendance (Ezaki, 2019). School-level factors include damaged or inaccessible classrooms due to political instability and natural disasters (Poyck, Koirala, Aryal, & Sharma, 2016), different language of instruction (Nepali in government and English in private institutions), and poor teaching and learning strategies, despite a high proportion of trained teachers (94% at primary and 78% at secondary levels) (Government of Nepal, 2014a, 2014b). A high student teacher ratio of 60:1 in primary and 102:1 in lower secondary (Government of Nepal, 2014a), mixed-ability and multi-grade classrooms result in inadequate guidance and monitoring of individual student achievement (Neupane, 2017).

Fourth, cross-sectoral policies are required to tackle maternal undernutrition, which is crucial in shaping the phenotype of offspring, and a deeper driver of children’s early growth and educational faltering, especially at pre-primary. Coordinated policy efforts may be effective, such as enforcement of a legal minimum marriage age and mandatory secondary education. Collectively, these efforts may help improve the status of women in society, thereby enhancing children’s full developmental and learning potential (Marphatia, Cole, Grijalva-Eternod, & Wells, 2016).

### 5.3 Limitations and strengths

There were several limitations to our study, some of which we were able to address. First, there may be a recall bias in pre-primary entry-age, but we validated its reliability by triangulating data on subsequent entry and progression. Second is the potential lack of precision in calculating over-age for grade attendance since children are likely to be more than one age during a school year or grade and the time-point in the school year when educational data on progression is collected. Third, mothers were generally unaware of children’s exam scores and we could not collect individual performance data from schools. However, grade repetition provided an indirect proxy of achievement. Fourth, we could not assess the effect of children’s cognitive development on their schooling because it was measured at age 12-years, at the same time as our primary outcome and later than our secondary outcome. Fifth, we did not measure maternal marriage age, but maternal education may provide an indirect proxy for marriage age as younger married women tend to complete less schooling. Sixth, self-reported land size is subject to measurement bias: size of small plots are generally over-estimated and large plots under-estimated (Dillon, Gourlay, McGee, & Oseni, 2019); estimating land size may be challenging for women who generally do not own land (Ministiry of Health & Population et al., 2012; Ministiry of Health & Population et al., 2017).

Despite these limitations, our study had several strengths. First, we used prospective longitudinal data on 797 mother-child dyads with a relatively good cohort retention rate at 12 years. Second, we had detailed data on maternal biological and social traits collected at recruitment into the RCT, children’s early growth patterns and their progression in school from pre-primary, the combination of which is not routinely collected by most studies. Third, we contributed to understanding education in lowland Nepal, a region which is still under-studied despite chronic over-age attendance. Fourth, we showed that factors acting in the years prior to school shape the age of pre-primary entry. Fifth, our models explained a substantial proportion of the variance in over-age attendance at 12 years and pre-primary entry-age.

## 6 Conclusion

Despite substantial grade slippage across LICs, both pre-primary entry-age and over-age/grade attendance are remarkably under-studied. Over-age attendance in lower secondary school is the product of a cumulative process of grade slippage from early in the educational trajectory. Late pre-primary entry starts a domino effect for subsequent grade faltering. We found that poor maternal nutritional status, parental lack of education, rural location and poor infant linear growth predicted late pre-primary entry. Some of these factors relate to maternal phenotype, which is shaped before children are even born. This suggests that efforts strengthening maternal factors may be most effective in ensuring children enter and progress through school at the expected age. In turn, education shapes these very factors, indicating the need for interdisciplinary efforts. Addressing nutrition, education and marriage age separately may in fact contribute to further inequality and perpetuate the inter-generational cycle of disadvantage.

## Declaration of Competing Interest

None.

## Figures and Tables

**Table A1:** Correlations of maternal and family components.^1^

	Maternal height (cm)	Maternal BMI (kg/m^2^)^2^	Maternal education (y)	Paternal education (y)	Material assets (score)	Land ownership (dhur)^2^	Parity (no. of births)
Maternal age	0.03	−0.01	-0.12**	-0.09*	−0.01	−0.06	0.64**
Maternal height		-0.10**	0.20**	0.13**	0.08*	0.16**	−0.03
Maternal BMI			0.10**	0.08*	0.11**	0.04	-0.07*
Maternal education				0.38**	0.46**	0.24**	-0.30**
Paternal education					0.32**	0.12**	-0.11**
Material assets						0.21**	-0.08*
Land ownership							-0.08*

1 Pearson coefficient.

2 BMI and land ownership were natural log-transformed for use in statistical tests.

* p ≤ 0.05.

** p ≤ 0.01.

## References

[bib0005] Adair L., Fall C., Osmond C., Stein A., Martorell R., Ramirez-Zea M. (2013). Associations of linear growth and relative weight gain during early life with adult health and human capital in countries of low and middle income: findings from five birth cohort studies. Lancet.

[bib0010] Alcott B., Rose P. (2017). Learning in India’s primary schools: How do disparities widen across the grades?. Int J Educ Dev..

[bib0015] Alderman H., Headey D.D. (2017). How Important is Parental Education for Child Nutrition?. World Dev..

[bib0020] Ashby J.A. (1985). Equity and Discrimination among Children: Schooling Decisions in Rural Nepal. Comp Educ Rev..

[bib0025] Ayral K. (2014). A Steep Climb Uphill: Does geography and environment create barriers to secondary-aged girls’ equitable access to, and participation in education in remote areas of Nepal?.

[bib0030] Berlinski S., Galiani S., Gertler P. (2006). The Effect of Pre-Primary Education on Primary School Performance.

[bib0035] Black M.M., Walker S.P., Fernald L.C.H., Andersen C.T., DiGirolamo A.M., Lu C. (2016). Early childhood development coming of age: science through the life course. The Lancet.

[bib0040] Brown J., Pollitt E. (1996). Malnutrition, poverty and intellectual development. Sci Am..

[bib0045] Brunette T., Crouch L., Cummiskey C., Dick A., Henny C., Jordan R. (2017). Repetition of Primary 1 and Pre-primary Education in Uganda.

[bib0050] Chen Q. (2015). Ready for school? Impacts of delayed primary school enrollment on children’s educational outcomes in rural China. Int J Educ Dev..

[bib0055] Cole T., Green P. (1992). Smoothing reference centile curves: the LMS method and penalized likelihood. Stat Med..

[bib0060] Cumming C., Acharya S., Moriani F., Shakya D., Levine V. (2012). Mid-term Evaluation of the School Sector Reform Program.

[bib0065] Devakumar D., Chaube S., Wells J., Saville N., Ayres J., Manandhar D. (2014). Effect of antenatal multiple micronutrient supplementation on anthropometry and blood pressure in mid-childhood in Nepal: follow-up of a double-blind randomised controlled trial. Lancet Glob Health.

[bib0070] Devakumar D., Fall C.H., Sachdev H.S., Margetts B.M., Osmond C., Wells J.C. (2016). Maternal antenatal multiple micronutrient supplementation for long-term health benefits in children: a systematic review and meta-analysis. BMC Med..

[bib0075] Dillon A., Gourlay S., McGee K., Oseni G. (2019). Land measurement bias and its empirical implications: Evidence from a validation exercise. Econ Dev Cult Change.

[bib0080] Dulal S., Liégeois F., Osrin D., Kuczynski A., Manandhar D.S., Shrestha B.P. (2018). Does antenatal micronutrient supplementation improve children’s cognitive function? Evidence from the follow-up of a double-blind randomised controlled trial in Nepal. BMJ Glob Health..

[bib0085] Engle P.L., Fernald L.C., Alderman H., Behrman J., O’Gara C., Yousafzai A. (2011). Strategies for reducing inequalities and improving developmental outcomes for young children in low-income and middle-income countries. The Lancet.

[bib0090] Ezaki N. (2019). Enrolment patterns of individual children left behind in the trend towards ‘quality education’: A case study of primary education in Nepal. Educ 3-13.

[bib0095] Fentiman A., Hall A., Bundy D. (2001). Health and cultural factors associated with enrolment in basic education: a study in rural Ghana. Soc Sci Med..

[bib0100] Fink G., Rockers P.C. (2014). Childhood growth, schooling, and cognitive development: further evidence from the Young Lives study. Am J Clin Nutr..

[bib0105] Glewwe P., Jacoby H. (1993). Living Standards Measurement Study Working Paper no. 98. Delayed primary school enrollment and childhood malnutrition in Ghana: An economic analysis.

[bib0110] Glewwe P., Jacoby H.G. (1995). An economic analysis of delayed primary school enrollment in a low income country: the role of early childhood nutrition. Rev Econ Stat..

[bib0115] Government of Nepal (2009). Flash 1 Report 2066 (2009-10).

[bib0120] Government of Nepal (2007). National Curriculum Framework for School Education in Nepal 2007.

[bib0125] Government of Nepal (2014). Flash I Report 2071 (2014-015).

[bib0130] Government of Nepal (2014). National Early Grade Reading Program (2014/15-2019/20).

[bib0135] Hammond C., Linton D., Smink J., Drew S. (2007). Dropout Risk Factors and Exemplary Programs: A Technical Report..

[bib0140] Halpern R., Myers R. (1986). Effects of early childhood intervention on primary school progress and performance in the developing countries. Comparative Education Review.

[bib0145] Hossain A. (2010). Age in Grade Congruence and Progression in Basic Education in Bangladesh. CREATE Pathways to Access.

[bib0150] Hungi N., Ngware M., Abuya B. (2014). Examining the impact of age on literacy achievement among grade 6 primary school pupils in Kenya. Int J Educ Dev..

[bib0155] Independent Evaluation Group (2015). Project Performance Assessment Report Nepal Education For All.

[bib0160] Jaganath D., Khatry S.K., Murray-Kolb L.E., LeClerq S.C., Christian P. (2015). The Role of Pre-Primary Classes on School-Age Cognition in Rural Nepal. J Pediatr..

[bib0165] Jha S., Lewin K. (2014). Delayed Entry in School and Human Capital Accumulation: Evidences from India. Indian J Hum Dev..

[bib0170] Kafle K., Jolliffe D., Winter-Nelson A. (2018). Do different types of assets have differential effects on child education? Evidence from Tanzania. World Dev..

[bib0175] Khanal S.K., Paudyal B.R., Dangal S. (2017). Early childhood development policies in Nepal: Achievements, learning, and implications. Early Childhood Education Policies in Asia Pacific.

[bib0180] Lewin K.M. (2007). Diversity in convergence: access to education for all. Comp. J. Comp. Int. Educ..

[bib0185] Lloyd C., Blanc A. (1996). Children’s Schooling in sub-Saharan Africa: The Role of Fathers, Mothers, and Others. Popul Dev Rev..

[bib0190] Lucas A., CIBA Foundation Symposium (1991). Programming by early nutrition in man. The Childhood Environment and Adult Disease.

[bib0195] MacKay D., Smith G., Dobbie R., Pell J. (2010). Gestational Age at Delivery and Special Educational Need: Retrospective Cohort Study of 407,503 Schoolchildren. PLoS Med..

[bib0200] Maluccio J., Hoddinott J., Behrman J., Martorell R., Quisumbing A., Stein A. (2009). The Impact of Improving Nutrition During Early Childhood on Education among Guatemalan Adults. Econ J..

[bib0205] March of Dimes, PMNCH, Save the Children, WHO (2012). Born Too Soon: The Global action report on preterm Birth.

[bib0210] Marphatia A.A., Reid A.M., Yajnik C.S. (2019). Developmental origins of secondary school dropout in rural India and its differential consequences by sex: A biosocial life-course analysis. Int J Educ Dev..

[bib0215] Marphatia A., Devakumar D., Wells J., Saville N., Reid A., Costello A. (2016). Maternal phenotype, independent of family economic capital, predicts educational attainment in lowland Nepalese children. Am J Hum Biol..

[bib0220] Marphatia A., Cole T., Grijalva-Eternod C., Wells J. (2016). Associations of gender inequality with child malnutrition and mortality across 96 countries. Glob Health Epidemiol Genomics.

[bib0225] Martorell R., Horta B., Adair L., Stein A., Richter L., Fall C. (2010). Weight Gain in the First Two Years of Life Is an Important Predictor of Schooling Outcomes in Pooled Analyses from Five Birth Cohorts from Low- and Middle-Income Countries. J Nutr..

[bib0230] Ministry of Education Nepal (2016). School Sector Development Plan, Nepal, 2016/17–2022/23.

[bib0235] Ministry of Education Nepal (2009). School Sector Reform Plan 2009-2015.

[bib0240] Ministry of Education Nepal, UNICEF, UNESCO (2016). Global Initiative on Out of School Children – Nepal Country Study.

[bib0245] Ministry of Health and Population Nepal, New ERA, ICF International (2012). Nepal Demographic and Health Survey 2011.

[bib0250] Ministry of Health and Population, Nepal, New ERA, ICF International (2017). Nepal Demographic and Health Survey 2016.

[bib0255] Moock P.R., Leslie J. (1986). Childhood malnutrition and schooling in the Terai region of Nepal. J Dev Econ..

[bib0260] Neupane P. (2017). Barriers to Education and School Attainment—Evidence from Secondary Schools in Rural Nepal. Int Educ Stud..

[bib0265] Nonoyama-Tarumi Y., Loaiza E., Engle P.L. (2010). Late Entry into Primary School in Developing Societies: Findings from Cross-National Household Surveys. Int Rev Educ..

[bib0270] Osrin D., Vaidya A., Shrestha Y., Baniya R., Manandhar D., Adhikari R. (2005). Effects of antenatal multiple micronutrient supplementation on birthweight and gestational duration in Nepal: double-blind, randomised controlled trial. Lancet.

[bib0275] Özaltin E., Hill K., Subramanian S. (2010). Association of Maternal Stature With Offspring Mortality, Underweight, and Stunting in Low- to Middle-Income Countries. JAMA J Am Med Assoc..

[bib0280] Poyck M.C., Koirala B.N., Aryal P.N., Sharma N.K. (2016). Joint Evaluation of Nepal’s School Sector Reform Plan Programme 2009-16.

[bib0285] Raj A., McDougal L., Silverman J.G., Rusch M.L.A. (2014). Cross-Sectional Time Series Analysis of Associations between Education and Girl Child Marriage in Bangladesh, India, Nepal and Pakistan, 1991-2011. PLOS ONE.

[bib0290] Reed B.A., Habicht J.P., Niameogo C. (1996). The effects of maternal education on child nutritional status depend on socio-environmental conditions. Int J Epidemiol..

[bib0295] Research Centre for Educational Innovation and Development (CERID) (2012). A Study on the Impact of Early Childhood Education and Development (ECED) Services in School Readiness in Nepal.

[bib0300] Rose P., Alcott B. (2015). How can education systems become equitable by 2030?.

[bib0305] Sabates R., Hossain A., Lewin K.M. (2013). School drop out in Bangladesh: Insights using panel data. Int J Educ Dev..

[bib0310] Sandjaja Poh B.K., Rojroonwasinkul N., Nyugen B.K.L., Budiman B., Ng L.O. (2013). Relationship between anthropometric indicators and cognitive performance in Southeast Asian school-aged children. Br J Nutr..

[bib0315] Schell L. (1997). Culture as a stressor: A revised model of biocultural interaction. Am J Phys Anthropol..

[bib0320] Seshie-Nasser H.A., Oduro A.D. (2016). Delayed primary school enrolment among boys and girls in Ghana. Int J Educ Dev..

[bib0325] Sharma D. (2016). How liberal is Nepal’s liberal grade promotion policy?.

[bib0330] Shrestha V., Shrestha R. (2017). Intergenerational effect of education reform program and maternal education on children’s educational and labor outcomes: Evidence from Nepal.

[bib0335] Smith L., Ramakrishnan U., Ndiaye A., Haddad L., Martorell R. (2003). The importance of women’s status for child nutrition in developing countries.

[bib0340] Stash S., Hannum E. (2001). Who goes to school? Educational stratification by gender, caste, and ethnicity in Nepal. Comp Educ Rev..

[bib0345] Stein A.D., Barros F.C., Bhargava S.K., Hao W., Horta B.L., Lee N. (2013). Birth Status, Child Growth, and Adult Outcomes in Low- and Middle-Income Countries. J Pediatr..

[bib0350] Sunny B.S., Elze M., Chihana M., Gondwe L., Crampin A.C., Munkhondya M. (2017). Failing to progress or progressing to fail? Age-for-grade heterogeneity and grade repetition in primary schools in Karonga district, northern Malawi. Int J Educ Dev..

[bib0355] UIS (2018). Nepal [Internet]. Nepal country snapshot. http://uis.unesco.org/country/np.

[bib0360] UIS, UNICEF (2015). Fixing the broken promise of education for all: Findings from the Global Initiative on Out-of-School Children.

[bib0365] UNDP (2018). Sustainable Development Goals [Internet]. http://www.undp.org/content/undp/en/home/sustainable-development-goals.html.

[bib0370] UNESCO (2017). Global Education Monitoring Report 2017/18. Accountability in Education. Meeting our commitments.

[bib0375] UNESCO (2010). Reaching the marginalized: EFA global monitoring report, 2010.

[bib0380] UNESCO UIS (2017). Metadata for the global and thematic indicators for the follow-up and review of SDG 4 and Education 2030.

[bib0385] Upreti N. (2016). Development during early childhood: pre-primary education in Nepal [Doctoral Dissertation].

[bib0390] Vaidya A., Saville N., Shrestha B., de L Costello A., Manandhar D., Osrin D. (2008). Effects of antenatal multiple micronutrient supplementation on children’s weight and size at 2 years of age in Nepal: follow-up of a double-blind randomised controlled trial. Lancet.

[bib0395] Wells J. (2010). Maternal capital and the metabolic ghetto: An evolutionary perspective on the transgenerational basis of health inequalities. Am J Hum Biol..

[bib0400] WHO (2011). Haemoglobin concentrations for the diagnosis of anaemia and assessment of severity. Vitamin and Mineral Nutritional Information System.

[bib0405] WHO Expert Consultation (2004). Appropriate body-mass index for Asian populations and its implications for policy and intervention strategies. Lancet.

[bib0410] WHO SEARO (1994). Multicentre study on low birth weight and infant mortality in India, Nepal and Sri Lanka.

[bib0415] WHO and UNICEF (2009). WHO child growth standards and the identification of severe acute malnutrition in infants and children. A Joint statement by the World Health Oranization and the United Nations Children's Fund.

[bib0420] Wils A. (2004). Late Entrants Leave School Earlier: Evidence from Mozambique. Int Rev Educ..

[bib0425] World Bank (1999). Project Appraisal Document for the Basic and Primary Education Programme.

